# Obesity-Associated Melanocortin-4 Receptor Mutations Are Associated With Changes in the Brain Response to Food Cues

**DOI:** 10.1210/jc.2014-1651

**Published:** 2014-07-25

**Authors:** Agatha A. van der Klaauw, Elisabeth A. H. von dem Hagen, Julia M. Keogh, Elana Henning, Stephen O'Rahilly, Andrew D. Lawrence, Andrew J. Calder, I. Sadaf Farooqi

**Affiliations:** University of Cambridge Metabolic Research Laboratories (A.A.v.d.K., J.M.K., E.H., S.O., I.S.F.), Wellcome Trust-Medical Research Council (MRC) Institute of Metabolic Science, Addenbrooke's Hospital, Cambridge CB2 0QQ, United Kingdom; MRC Cognition and Brain Sciences Unit (E.A.H.v.d.H., A.D.L., A.J.C.), Cambridge CB2 7EF, United Kingdom; and School of Psychology (A.D.L.), Cardiff University, Cardiff CF10 3XQ, United Kingdom

## Abstract

**Context:**

Mutations in the melanocortin-4 receptor (MC4R) represent the commonest genetic form of obesity and are associated with hyperphagia.

**Objective:**

The aim of this study was to investigate whether melanocortin signaling modulates anticipatory food reward by studying the brain activation response to food cues in individuals with MC4R mutations.

**Design/Setting/Participants/Main Outcome Measure:**

We used functional magnetic resonance imaging to measure blood oxygen level-dependent responses to images of highly palatable, appetizing foods, bland foods, and non-food objects in eight obese individuals with MC4R mutations, 10 equally obese controls, and eight lean controls with normal MC4R genotypes. Based on previous evidence, we performed a region-of-interest analysis centered on the caudate/putamen (dorsal striatum) and ventral striatum.

**Results:**

Compared to non-foods, appetizing foods were associated with activation in the dorsal and ventral striatum in lean controls and in MC4R-deficient individuals. Surprisingly, we observed reduced activation of the dorsal and ventral striatum in obese controls relative to MC4R-deficient patients and lean controls. There were no group differences for the contrast of disgusting foods with bland foods or non-foods, suggesting that the effects observed in response to appetizing foods were not related to arousal.

**Conclusion:**

We identified differences in the striatal response to food cues between two groups of obese individuals, those with and those without MC4R mutations. These findings are consistent with a role for central melanocortinergic circuits in the neural response to visual food cues.

Mutations that disrupt signaling through the melanocortin 4 receptor (MC4R) represent the commonest highly penetrant genetic form of obesity, being found in 2–5% of severely obese individuals (1). MC4Rs are widely expressed in the hypothalamus, brainstem, and other brain regions, where they mediate the anorectic response to the adipocyte-derived hormone leptin and the satiety response to gut hormones such as peptide YY and ghrelin (2). MC4Rs are also expressed in dopamine-rich regions of the striatum (3), and there is a growing body of evidence in rodents to suggest that melanocortin signaling modulates food reward (4). To investigate the impact of genetic disruption of central melanocortin signaling on the brain response to anticipatory food reward in humans, we studied obese individuals with heterozygous mutations in MC4R that completely disrupt melanocortin signaling in cells and are associated with increased food intake (1). We used functional magnetic resonance imaging (fMRI) to measure blood oxygen level-dependent responses to images of highly palatable, appetizing foods, bland foods, and non-food objects in eight MC4R-deficient individuals and in 10 equally obese and eight lean controls in whom MC4R mutations had been excluded.

## Subjects and Methods

### Participants

MC4R mutation carriers were identified by direct nucleotide sequencing (4). We identified eight MC4R-deficient patients with heterozygous mutations that had previously been shown to result in a complete loss of function in vitro by measuring cAMP production (1). MC4R mutations were excluded in the two controls groups—10 overweight/obese controls and eight lean controls. Details on the study participants are given in Tables 1 and 2. All subjects were weight stable for at least 3 months, were not taking any medication (healthy volunteers), were right-handed, and had no history of psychiatric disease. The study was approved by the Local Regional Ethics Committee in Suffolk. All subjects provided written informed consent. Motivational state (eg, hunger) modulates responses to food-related stimuli; we therefore studied all subjects in the satiated state. All participants received a standardized breakfast and lunch before the study at fixed times. Meals were calculated to provide 20 and 35% of daily energy requirements, respectively. Energy requirements were calculated with the Schofield formula (5), which allows for the different energy requirements of lean and obese individuals. The macronutrient content was 50% carbohydrate, 30% fat, and 20% protein. The fMRI scan was performed exactly 1 hour after consumption of the lunch in all instances. Hunger or fullness visual analog scores taken just before participants went into the fMRI scan were not different between the three groups (lean controls—hunger, 2.0 ± 0.4; fullness, 6.5 ± 0.7; obese controls—hunger, 2.7 ± 0.6; fullness, 5.4 ± 0.8; MC4R—hunger, 2.4 ± 0.5; fullness, 5.6 ± 0.7; hunger, *P* = .624; fullness, *P* = .551).

**Table 1.** Characteristics of Groups

Group	Gender, M/F	Age Range, y	BMI (mean ± SEM), kg/m^2^
MC4R-deficient participants	5/3	18–47	34.5 ± 7.3; range 26–44
Obese participants	4/6	31–50	33.2 ± 5.4; range 28–41
Lean participants	2/6	19–54	22.4 ± 1.9; range 21–25

**Table 2.** Mutations of Subjects

Subject	Mutation
1	I125K, related to subjects 5 and 8
2	Y35X;D37V, related to subject 3
3	Y35X;D37V, related to subject 2
4	G252S
5	I125K, related to subjects 1 and 8
6	R236C
7	R165W
8	I125K, related to subjects 1 and 5

### Experimental design

While lying in the scanner, participants viewed images of appetizing (eg, chocolate cake), disgusting (eg, rotten meat), and bland foods (eg, uncooked rice), plus non-food household objects (30 unique items in each category). To control for the possibility that areas engaged by appetizing foods reflect increased emotional arousal, we included a disgusting food category matched to the appetizing foods on rated arousal (6). Images of appetizing and disgusting food images were preselected to be equal in rated arousal (6). Color images were selected from the International Affective Picture Series, supplemented by copyright-unrestricted images obtained from the internet. All images were cropped to be of the same size. During the fMRI experiment, stimuli were presented in 18-second blocks, with each block containing 10 images from the same category. Each image was displayed for 1300 milliseconds, followed by a 500-millisecond fixation screen. The four block types were presented in random order, with nine blocks for each category. Stimuli were viewed via an angled mirror above the participants' eyes, which reflected images back-projected from a translucent screen positioned in the bore of the magnet to the rear of the participants' head. Images were shifted randomly, slightly to the left or right of the center of the screen (1.7° visual angle). Participants were asked to respond by button press whether the image was presented to the left or right of the center of the screen.

### fMRI data acquisition

Functional imaging data were acquired using a 3T Tim Trio (Siemens) scanner. Whole-brain T2*-weighted echo planar images (EPIs) were acquired with a repetition time of 2000 milliseconds, echo time of 30 milliseconds, flip angle of 78º, and 32 axial oblique slices with 3-mm isotropic resolution. A total of 336 volumes were acquired, for a total imaging time of 11 minutes 12 seconds. A high-resolution structural MP-RAGE scan for normalization purposes was also acquired (voxel size, 1 × 1×1 mm; repetition time, 2250 ms; echo time, 2.98 ms; inversion time, 900 ms; flip angle, 9°; total scan time, 4 min 16 s).

### fMRI analysis

Data were analyzed using SPM5 (Wellcome Trust Centre for Neuroimaging, www.fil.ion.ucl.ac.uk/spm). The first six volumes were discarded to allow for equilibration effects. The EPIs were corrected for slice time differences and realigned to the first scan by rigid body transformations to correct for head movements. EPI and structural scans were coregistered and normalized to the T1 standard template in Montreal Neurological Institute space (MNI-International Consortium for Brain Mapping) and smoothed with a Gaussian kernel of 8 mm FWHM. Data were analyzed using general linear models within SPM5. At the first level, condition effects were estimated using boxcar regressors convolved with the canonical hemodynamic response function, and movement parameters were included as regressors to account for residual movement-related variance. Data were high-pass filtered (128 s) to remove low-frequency signal drift. Statistical parametric maps were then generated for each individual subject by estimating activation contrasts between conditions (eg, appetizing vs bland). To determine group differences at the second level, we set up an ANOVA at the whole-brain level for each contrast and conducted a region-of-interest (ROI) analysis. Based on strong evidence for the involvement of dorsal and ventral striatum in food-reward processing, and more specifically in obesity, our primary ROIs were 8-mm radius spheres centered on the caudate/putamen (dorsal striatum) (x = −15, y = 18, z = 12) (7) and ventral striatum (x = −8, y = 16, z = −12) (6). Corrections for multiple comparisons were performed by small volume correction using a Bonferroni correction (family-wise error).

An additional exploratory analysis of other brain regions that have also been implicated in reward and in obesity, including amygdala, orbitofrontal cortex, and somatosensory cortex, as well as the insula/frontal operculum in disgust processing, was performed using the more lenient threshold of *P* < .001 uncorrected. For completeness, we also performed a whole-brain analysis for all contrasts at the uncorrected *P* < .001 level, with an extent threshold of 10 voxels (data shown in Supplemental Table 1). Significant or borderline group main effects in our ROIs were followed up with post hoc *t* tests between each of the groups.

## Results

Images of appetizing foods shown to individuals undergoing fMRI scanning are associated with the activation of specific brain regions involved in a reward network that includes the striatum, amygdala, ventral tegmental area, and orbitofrontal and prefrontal cortex, which are the source and target of dopaminergic neurons that mediate food reward (8). In view of the limited number of MC4R-deficient individuals who conformed to our strict inclusion criteria and were thus available for this study, we performed a ROI analysis. Based on evidence for the involvement of dorsal and ventral striatum in food-reward processing, our primary regions of interest were the dorsal and ventral striatum. We first established group differences in the neural response to viewing appetizing foods in the dorsal and ventral striatum.

**Figure 1.**
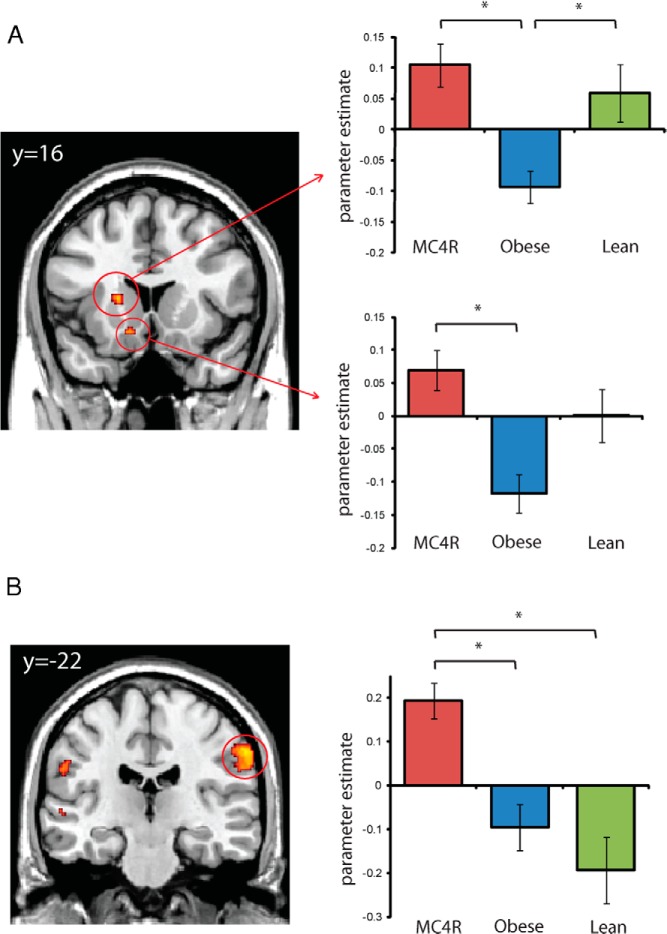
Activation of dorsal and ventral striatum (A) and somatosensory cortex (B) in response to appetizing foods compared to non-food objects in MC4R deficiency and obese and lean controls. Coronal sections show the main effect of group at *P* < .005 uncorrected for display purposes. Extracted data plotted for each group are the average group parameter estimates at the peak voxel for ventral (x = −12, y = 18, z = −12) and dorsal striatum (x = −18, y = 14, z = 6) after multiple comparisons correction (small-volume correction), and at *P* < .001 uncorrected for the somatosensory cortex (x = 64, y = −20, z = 38). Error bars represent the standard error of the mean.

A comparison of appetizing foods compared to non-foods produced a significant difference between the groups in the left dorsal striatum (caudate-putamen) [F(2,23) = 9.92; *P* = .04 small-volume correction (svc)] (Figure 1A and Table 3), reflecting reduced activation in obese controls relative to MC4R-deficient patients and lean controls (MC4R, T = 4.18, *P* = .01 svc; lean controls, T = 3.50, *P* = .02 svc). The dorsal striatum showed a similar group effect to appetizing foods vs bland foods [F(2,23) = 7.33; *P* = .07 svc], reflecting a significantly reduced response in the obese group compared with the MC4R-deficient patients and lean controls (MC4R—T = 3.81, *P* = .01 svc; lean controls—T = 3.58, *P* = .03 svc). A similar pattern was also apparent in the left ventral striatum [F(2,23) = 9.25; *P* = .05 svc] (Figure 1A), where obese controls showed reduced response to appetizing foods vs non-food objects relative to the MC4R-deficient group (T = 4.25; *P* = .008 svc) and a similar trend relative to the lean controls. For each of these contrasts, the MC4R group did not significantly differ from lean controls. There were no group differences in dorsal or ventral striatum for the contrast of disgusting foods with bland foods or non-foods, suggesting that the effects observed in response to appetizing foods were not related to arousal more generally.

**Table 3.** fMRI Striatal ROI Analysis in MC4R-Deficient Individuals, Lean and Obese Controls

Brain Region	*P* (FWE)	F or T	MNI Coordinates		
			x	y	z
Appetizing > non-food					
Dorsal striatum left (caudate/putamen)					
Main effect of group	.04	F = 9.92	−18	14	6
MC4R > obese	.01	T = 4.18	−18	14	6
LC > obese	.02	T = 3.45	−20	14	8
Ventral striatum left					
Main effect of group	.05	F = 9.25	−12	18	−12
MC4R > obese	.01	T = 4.25	−12	20	−12
LC > obese	.10	T = 2.87	−12	16	−12
Appetizing > bland					
Dorsal striatum left (caudate/putamen)					
Main effect of group	.07	F = 7.33	−14	20	16
MC4R > obese	.01	T = 3.81	−12	20	16
LC > obese	.03	T = 3.26	−16	18	8

Abbreviations: LC, lean controls; FWE, family-wise error; MNI, Montreal Neurological Institute. Statistics and coordinates for analysis of group differences in striatal ROIs (small volume corrected for multiple comparisons). Main effects of group are listed, as well as post hoc *t* tests.

**Figure 2.**
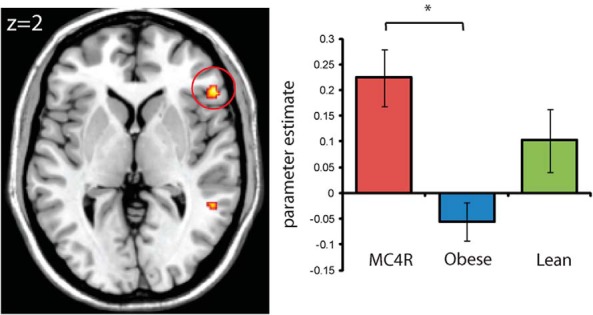
Significant differences in activation in right frontal operculum (x = 54, y = 30, z = 2) in response to disgusting foods compared to non-foods in MC4R deficiency and obese and lean controls. Axial section shows the main effect of group at *P* < .005 uncorrected for display purposes. Extracted data plotted for each group are the average group parameter estimates at the peak voxel. Error bars represent the standard error of the mean.

Our additional exploratory analyses of the amygdala, orbitofrontal cortex, and somatosensory cortex, at a more lenient threshold of *P* < .001 uncorrected, revealed group differences in response to disgusting foods relative to non-foods in the right frontal operculum (x = 54, y = 30, z = 2) (Figure 2 and Supplemental Table 1), where MC4R-deficient individuals showed significantly greater response than obese controls (T = 4.88; *P* < .001), but no difference from lean controls. The frontal operculum is involved in processing taste information and relating it to motivation, emotion, and visceral activity (9). Studies in rodents indicate that forebrain MC4R stimulation influences taste responsiveness, potentially by modulation of the perceived intensity of taste stimuli (10, 11). In addition, we found significantly greater activity in the MC4R-deficient group [F(2,23) = 11.13; *P* < .001 uncorrected) for appetizing foods vs non-foods compared to obese (T = 3.70; *P* = .001) and lean controls (T = 4.43; *P* < .001) in the inferior parietal cortex (x = 62, y = −22, z = 40), which corresponds to the somatosensory cortex area that encodes sensation in the mouth, lips, and tongue (Figure 1A). We found no group differences in the amygdala or orbitofrontal cortex for any of the contrasts, even at our more lenient threshold of *P* < .001 uncorrected. Additional brain regions that showed a main effect of group at the whole brain uncorrected *P* < .001 level for each contrast are listed in Supplemental Table 1.

Differences in brain activation were not explained by differences in reward sensitivity between the groups as assessed by the Behavioral Activation Scale-drive scale (12) (F < 1; *P* = .8) which has previously been associated with variability in responses to food reward (6). There were also no group differences in how participants rated the images on pleasantness (F < 1; *P* = .57) and disgust (F < 1; *P* = .23) postscanning.

## Discussion

In this small study, we have been able to distinguish the brain response to food images between two highly comparable groups of obese people with and without genetic disruption of melanocortin signaling. In our study, all participants were studied in the satiated state to adjust for potential differences in motivational state between the groups (hunger ratings using visual analog scores were comparable before scanning).

Several studies have reported changes in striatal activation in response to food-related stimuli in obese individuals. Some have proposed that a hyper-responsiveness of reward circuitry, when presented with food images, increases the risk for overeating (13, 14). Others hypothesize that obese individuals show hyporesponsiveness of reward circuitry after the consumption of food, which leads them to overeat to compensate for this deficiency (15). In a prospective study, weight gain was associated with a reduction in striatal activation in response to palatable food intake relative to baseline response (16). Our findings suggest that some obese individuals show hyporesponsivity of reward circuitry to visual food cues when studied in the satiated state (which may more closely parallel studies of food consumption). Our findings do not preclude that increased striatal activation may be seen in some obese individuals in response to food cues presented in the fasted/hungry state. Of note, in many studies, participants were studied after fasting for several hours, and motivational state may have modulated the striatal response to images of food and/or consumption of food. Our findings argue against a model where chronic overconsumption is sufficient to account for decreased striatal activation. Instead, our findings suggest that differences in brain responses to food cues in obese people are more likely to be due to differences in neural circuitry that occur with weight gain. We suggest that these neural changes require intact melanocortin signaling and, as such, are not seen in individuals with mutations that disrupt MC4R.

It has been suggested that differences in striatal activation may reflect differences in dopaminergic tone. Obese rats, relative to lean rats, show reduced D2 receptor density in the hypothalamus and in the striatum, and chronic excessive intake of high-calorie foods and attendant weight gain results in down-regulation of postsynaptic D2 receptors, increased D1 receptor binding, and decreased D2 sensitivity in animals (17, 18). Obese vs lean humans show reduced striatal D2 receptor density measured by positron emission tomography imaging in some studies (19). How might disruption of melanocortinergic signaling modulate dopaminergic circuits involved in food reward? Several studies in rodents have demonstrated that injection of melanocortins or of the endogenous MC4R antagonist, Agouti-related peptide, can modulate dopamine-containing neurons within brain regions known to mediate reward-based decision making and augment operant responding for palatable food (3). However, the direction of response seen varies depending on the anatomical site of injection, suggesting that multiple excitatory and inhibitory inputs on MC4R-expressing neurons are likely to contribute to the regulation of dopamine turnover. Interestingly, a recent case report suggested that pharmacological modification of dopaminergic tone by methylphenidate might stimulate weight loss in a patient with MC4R deficiency (20).

The explanation for increased activation of oral somatosensory cortex in the MC4R-deficient group is unclear. Pharmacological studies in rodents suggest that melanocortins do not modulate taste (as measured by licking frequency for sucrose/quinine). It is plausible that increased oral somatosensory cortex activation in MC4R-deficient humans may reflect increased anticipatory orosensory activity or salivation in response to appetizing food images in this group, although this was not formally tested. Increased somatosensory cortex activation has been observed in response to food reward (but not to monetary reward) in some previous studies (21). Using positron emission tomography, obese people showed greater resting metabolic activity in the oral somatosensory cortex relative to lean people, and using fMRI, obese vs lean adolescents exhibited greater activation in the oral somatosensory cortex in response to receipt of a chocolate milkshake vs receipt of a tasteless solution (22). Interestingly, apart from the somatosensory cortex findings in our exploratory analysis, the brain response of the MC4R-deficient individuals was not different from lean volunteers. This might be due to compensatory mechanisms related to the signaling defect in MC4R deficiency or the possibility that any changes might be too subtle to detect with fMRI. Alternatively, we recognize that the sample size is small due to the nature of the condition studied, and these findings will have to be repeated in larger studies. Nonetheless, our findings from this preliminary study suggest that central melanocortin signaling is involved in modulating the neural response to food cues in human obesity. Further studies will be needed to investigate the precise neural circuits that explain these observations. Understanding the complex interplay of biological and behavioral factors involved in the response to rewarding food cues may suggest interventions aimed at limiting the overconsumption of highly palatable foods associated with weight gain.
